# Health-related quality of life among long-term (≥5 years) prostate cancer survivors by primary intervention: a systematic review

**DOI:** 10.1186/s12955-017-0836-0

**Published:** 2018-01-23

**Authors:** Salome Adam, Anita Feller, Sabine Rohrmann, Volker Arndt

**Affiliations:** 1 Division of Chronic Disease Epidemiology, Epidemiology, Biostatistics and Prevention Institute, University of Zurich, Zurich, Switzerland; 2 National Institute for Cancer Epidemiology and Registration (NICER), University of Zurich, Zurich, Switzerland; 3 Unit of Cancer Survivorship, Division of Clinical Epidemiology and Aging Research, German Cancer Research Center (DKFZ), Heidelberg, Germany

**Keywords:** Prostate cancer, Long-term effects, Health-related quality of life, Survivors, Systematic review, Intervention

## Abstract

**Background:**

Due to an improving prognosis, and increased knowledge of intervention effects over time, long-term well-being among prostate cancer (PC) survivors has gained increasing attention. Yet, despite a variety of available PC interventions, experts currently disagree on optimal intervention course based on survival rates.

**Methods:**

In January 2017, we searched multiple databases to identify relevant articles. Studies were required to assess at least two different dimensions of health-related quality of life (HRQoL) in PC survivors ≥5 years past diagnosis with validated measures.

**Results:**

Identified studies (*n* = 13) were mainly observational cohort studies (*n* = 10), conducted in developed countries with a sample size below 100 per study arm (*n* = 6). *External-beam radiation therapy* was the most common intervention (*n* = 12), whereas only three studies included patients on *active surveillance* or on *watchful waiting*.

Studies were largely heterogeneous in cancer stage at diagnosis, intervention groups and instruments. All identified studies either used the EORTC QLQ-C30 (*n* = 5) or the SF-36 (*n* = 7) to assess generic HRQoL, yet 11 different instruments were employed to assess PC specific urinary, bowel and sexual symptoms. Overall, no consistent pattern between intervention and HRQoL was observed. Results from two randomized-controlled-trials (RCTs) and one observational study, comparing HRQoL by primary intervention in localized PC survivors suggest that long-term HRQoL does not differ by intervention. However, observational studies that included a combination of localized and locally advanced stage PC survivors identified HRQoL differences for various scales including physical well-being, social and role function, vitality, and role emotional.

**Conclusion:**

This review reveals the number of publications comparing HRQoL by primary intervention in long-term PC survivors is currently limited. Robust data from two RCTs and one observational study suggest that HRQoL does not seem to differ by intervention. However, the heterogeneity of studies’ methodologies and results hindered our ability to draw a clear conclusion. Therefore, in order to answer the question of which primary intervention is superior with respect to long-term HRQoL in PC patients, more high-quality, large-scale prospective cohort studies, or RCTs with repeated HRQoL assessments, are urgently needed.

**Electronic supplementary material:**

The online version of this article (10.1186/s12955-017-0836-0) contains supplementary material, which is available to authorized users.

## Background

In economically developed countries, prostate cancer (PC) continues to be the most frequent cancer in men [1]. In Europe, for example, approximately 400,000 men are diagnosed with PC annually [2]. Patient prognosis has substantially improved due to earlier diagnosis and advancements in therapy, leading to five-year relative survival rates of 99.1% (2008) in the US [3] and 93% in Europe [4]. Consequently, the number of PC survivors is on the rise [5]. In particular, the number of long-term survivors (i.e. those still alive 5 years after initial diagnosis [6]) is substantially increasing.

A variety of intervention options, including *radical prostatectomy* (RP), *radiotherapy* (*external beam* (EBRT) or *brachytherapy* (BT)), *chemotherapy* (CT), *cyberknife* (CK), *cryotherapy* (CRYO), *androgen deprivation therapy* (ADT), *active surveillance* (AS) and *watchful waiting* (WW)) are now available. [7–10] However, there is currently no agreement on the optimal intervention, based on survival rates, especially for men with localized stage PC [8, 9, 11].

Despite increased awareness regarding long-term outcomes and patient-reported outcomes (including health-related quality of life (HRQoL)), a gold-standard definition of HRQoL does not currently exist. However, researchers agree that HRQoL is a multidimensional concept that encompasses all aspects of survivors’ well-being including physical, psychological, social and spiritual health [12, 13]. Additionally, global HRQoL (or overall health perceptions) must be added to this multidimensional concept, as it has proven to be an important predictor of individuals’ health [14].

Although HRQoL outcomes are useful to define the harmful and beneficial effects of interventions from the patient’s perspective, differences in HRQoL outcomes of long-term PC survivors (≥ 5 years since diagnosis) [15] between interventions have rarely been documented [16, 17]. Due to high PC survival rates and low PC-specific mortality rates (which do not differ between interventions [8, 18]), information on long-term HRQoL should be analyzed and subsequently considered as an additional factor in intervention decisions. HRQoL is especially relevant because other measurements (e.g. survival/mortality rates) do not currently indicate superiority of one intervention over the others [11, 19–21].

This systematic review aims to identify all studies assessing HRQoL among long-term PC survivors by primary intervention. Findings will be synthesized and critically discussed with respect to study design and methodology.

## Method

We followed the standard systematic review methodology outlined by the Centre for Reviews and Dissemination (York, UK) [22] and the Preferred Reporting Items for Systematic Reviews and Meta-Analysis (PRISMA) group [23].

### Study eligibility criteria

This systematic review includes all quantitative comparative studies on PC survivors diagnosed a minimum of 5 years prior to HRQoL assessment. When studies also included short- or medium-term survivors, it was critical the researchers of these studies examined results specifically pertaining to long-term PC survivors.

At minimum, study outcomes had to report on overall/general HRQoL plus one HRQoL domain, or at least two HRQoL domains. Domains were defined as physical, psychological, social and spiritual well-being [12, 24]. Only validated assessment instruments were included, such as the European Organization for Research and Treatment of Cancer Core Questionnaire (EORTC QLQ-C30) [25], the 36-item Short Form Health Survey (SF-36) [26] or the Functional Assessment of Cancer Therapy - General (FACT-G) [27]. Further, we required HRQoL results to be explicitly reported by type of primary intervention. Interventions could be either RP, EBRT, BT, ADT, CT, CK, CRYO, AS or WW, as well as, combinations of these interventions. It was necessary each study compared the HRQoL of different interventions, or one intervention to the HRQoL of a reference group (e.g. general population). Without an available gold-standard classification of intervention options (e.g. active surveillance), all intervention options are classified as “intervention,” for our purposes [28–30]. Moreover, researchers had to report on information regarding age and date of diagnosis and time post diagnosis. All included articles were published in English, German, French or Italian.

### Search strategy and study selection

The literature search was completed in January 2017 using the following electronic databases: Pubmed, Medline, Embase, PsychInfo, Cinahl, Web of Science and Cochrane Central Register of Controlled Trials. Additionally, we hand-searched the bibliographies of reviews, conference proceedings, and supplements to identify further relevant studies. Authors of these publications were contacted for further details.

The following combinations were used: *“quality of life, HRQoL, patient satisfaction, well-being, general health status assessment, qlq c30, pr 25, sf 36”* AND *“cancer survivor, long-term, year after”* AND *“prostate cancer, prostate adenocarcinoma, prostate neoplasm, prostate neoplasia, prostate carcinoma”* (Additional file 1: Appendix A).

**Fig. 1:**
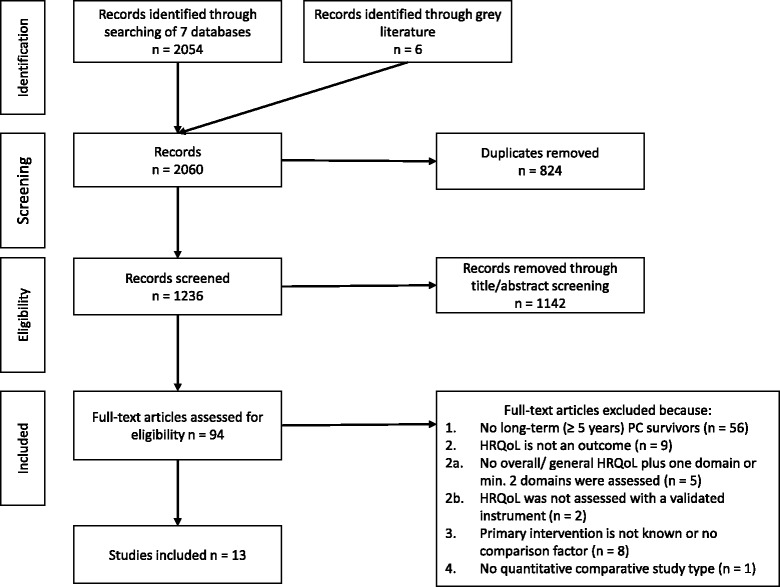
Flow diagram of the search and selection process

One author (SaA) assessed eligibility and selected the articles by screening records based on title/abstract review. Further, two reviewers (SaA and AF) assessed the full-texts according to predefined, hierarchically ordered inclusion and exclusion criteria. In the case of doubt, a third reviewer (VA) made the final decision. The flow diagram of the search and selection process is outlined in Fig. 1.

### Data extraction and quality assessment

Data were independently extracted by two reviewers (SaA, AF) using a systematic scheme containing the following study characteristics: title, first author, year, country, study design, age range, cancer stage, intervention(s), comorbidities, response rate, time since diagnosis/randomization, HRQoL instrument(s), statistical methods and results. Only data pertaining to long-term survivors was extracted. Reviewers described study results and indicated whether they were statistically significant and/or clinically meaningful [31–33]. The same reviewers assessed the methodological quality of each article, following the risk of bias (RoB) criteria based on the GRADE approach [34], with the following additional criteria: adjustment for attrition error, sample size power, control for confounding, reporting of results appropriate (plots/diagrams/tables printed sufficiently, lack of selective reporting of results), statistical significance test(s) performed and baseline data available.

## Results

### Literature search results

Two thousand sixty articles were identified through the literature search. After removing duplicates, 1236 articles remained. Screening of titles and abstracts identified 94 potentially eligible articles (Figure 1). Full-text analyses identified 13 articles, which were included in data extraction [35–47].

### Study characteristics

Studies were exclusively conducted in developed countries: seven in Europe [35, 37–39, 41, 43, 47], three in Japan [44–46], two in the US [40, 42] and one completed in the US and Europe [36] (Table 1). The majority were observational prospective cohort studies (*n* = 7) [35, 38, 40, 42, 44–46], three were observational retrospective cohort studies [39, 43, 47] and three were randomized controlled trials (RCTs) [36, 37, 41] (Tables 1 and 2).

**Table 1:** Characteristics of included studies

		At survey ≥5 years		Mean/ Median (Range)^a^		At diagnosis^g^
First Author/ Year, Country	Study Design	Sample Size (*n*)	Intervention (%)	Age at survey (years)	Follow-up time^f^ (years)	Cancer Stage (%)
Berg, A/ 2007, Norway [35]	Hospital-based observational prospective monocentric cohort study	64	EBRT (100) [+ADT (44.0)]^e^	66^c^ (48-81)	11 (10-16)	Localized PC (33.0) Locally advanced PC (67.0)
Brundage, M/ 2015, UK and US [36]	Hospital-based mulitcentric randomized controlled trial	85-111^d^	1. ADT (50.0)^c^ 2. ADT + EBRT (50.0)^c^	69.7^c^ (65.5 −73.5)	(5-8)	Locally advanced PC (100.0)
Donovan, J L / 2016, UK [37]	Population-based multicentric randomized controlled trial	1413-1463^d^	1. AS (33.2) 2. RP (33.7) 3. EBRT (33.1)	62^c^	(5-6)	Localized PC (100.0)
Fransson, P/ 2008, Sweden [38]	Hospital-based observational prospective monocentric cohort study	64	1. EBRT (42.2) + ADT (20.3) 2. Controls (57.8)	78.1 (62-87)	14.7 (13.5 – 16.4)	Localized PC (89.9) Locally advanced PC (11.1)
Fransson, P/ 2009, Sweden [39]	Hospital-based observational monocentric retrospective cohort study	54	1. EBRT (50.0) 2. WW (50.0)	78 (54 – 88)	9.6 (6.4-16.3)	Local PC (100.0)
Galbraith, M E/ 2005, US [40]	Hospital-based observational prospective monocentric cohort study	137	1. WW (11.5)^c^ 2. RP (21.4)^c^ 3. EBRT – C (9.9)^b,c^ 4. EBRT - PB (11.5)^b,c^ 5. EBRT - MB (20.3)^b,c^ 6. EBRT -LD (13.7)^b,c^ 7. EBRT - HD (17)^b,c^	69.9^c^	5.5	No information
Giberti, C/ 2009, Italy [41]	Hospital-based monocentric randomized controlled trial	174	1. RP (44.5) 2. BT (55.5)	65.3^c^ (56-74)^c^	5	Localized PC (100.0)
Johnstone, P A S/ 2000, US [42]	Hospital based observational monocentric prospective cohort study	46	EBRT (100.0) [+ ADT (43.5)]^e^	80 (62-90)	13.9 (10 - 23)	Localized PC Locally advanced PC
Mols, F/ 2006, Denmark [43]	Population-based observational retrospective cohort study	780	1. RP (32.9) 2. EBRT (41.4) 3. ADT (13.7) 4. EBRT (11.9)	75	(5-10)	Localized PC (76.0) Locally Advanced PC (18.0) Unknown (6.0)
Namiki, S/ 2011, Japan [44]	Hospital-based observational prospective monocentric cohort study	111	1. RP (43.2) + ADT (48) 2. EBRT (56.8) + ADT (100.0)	69.5^c^ (53 – 84)	5	Locally Advanced PC (100.0)
Namiki, S/ 2014, Japan [45]	Hospital-based observational prospective monocentric cohort study	91	RP (100.0)	63.9^c^	8.5 (7.1 - 10.25)	Localized PC (94.5) Locally Advanced PC (5.5)
Shinohara, N/ 2013, Japan [46]	Hospital-based observational monocentric prospective cohort study	67	1. EBRT (32.4) 2. RP (67.6)	68^c^ (53-79)	5	Localized PC (93.4) Locally Advanced PC (6.6)
Thong, M S/ 2010, Nether-lands [47]	Population-based observational retrospective cohort study	142	1. AS (50.0) [+ ADT (2.8)/ +RP (1.4)/ + EBRT (7)/ + EBRT + ADT (1.4)]^e^ 2. EBRT (50.0) + [RP (7)/ + ADT (2.8)/ + EBRT (1.4) + EBRT + ADT (1.4)]^e^	75.8	7.8	Localized PC (100.0)

*RP* Radical Prostatectomy, *EBRT* External Beam Radiotherapy (refers to the external delivery of any type of radiation), *BT* Brachytherapy, *WW* Watchful Waiting, *AS* Active Surveillance, *ADT* Androgen Deprivation Therapy

^a^Mean/Median for total sample

^b^EBRT-C — Conventional radiation; EBRT-HD — High-dose mixed-beam radiation; EBRT-LD — Low-dose mixed-beam radiation; EBRT-MB — Standard protocol/mixed-beam radiation; EBRT-PB — Proton beam radiation

^c^Sample size/Age at enrolment in study or randomization

^d^Sample sizes at different time points ≥ 5 years

^e^Secondary intervention(s)

^f^Either time since diagnosis or time since randomization

^g^Categorization: local PC – T1 & T2, locally advanced PC T3 & T4

**Table 2:** Summary table of study characteristics

Characteristic						Frequency
Study Design	Randomized controlled trial Observational prospective cohort study Observational retrospective cohort study					3 7 3
Recruitment	Monocentric hospital-based Multicentral hospital-based Population-based					9 1 3
Comparison: intervention vs. general population^a^	RP	EBRT	ADT	WW	AS	
	X					2
		X^b^				5
			X			1
				X		1
					X	1
Comparison: intervention vs. intervention^a^	RP	EBRT	ADT	WW	AS	
	X	X			X	1
	X	X^d^				1
	X	X				1
		X vs. X^c^				1
		X^c^	X			1
		X			X	1
		X		X		1
	X	X	X	X		1
	X	X^e^		X		1
	X	X^f^				1
Sample sizes (total population)	<100 101 – 200 780 1463 (after 5 years since randomization) respectively 1413 participants (6 years since randomization)					6 5 1 1
Years since diagnosis/ randomization	Long-term survivors (5-10 years after diagnosis) Very long-term survivors (10 + years after diagnosis)					10 3
Stage at diagnosis	Localized (T1/T2) PC Locally advanced (T3/T4 any N1/M1) PC Localized & locally advanced PC No information					3 2 7 1
Recurrent PC survivors	No information Excluded Included					10 1^g^ 2
Progressive PC survivors	No information Excluded Included					5 3 5

^a^Some studies had multiple comparisons

^b^“Plus ADT and/or clinical progression”

^c^Plus ADT

^d^Brachytherapy

^e^EBRT-C — Conventional radiation; EBRT-HD — High-dose mixed-beam radiation; EBRT-LD — Low-dose mixed-beam radiation; EBRT-MB — Standard protocol/mixed-beam radiation; EBRT-PB — Proton beam radiation

^f^Brachytherapy

^g^Excluded because they died

#### Recruitment of survivors

Recruitment was monocentric hospital-based in nine studies [35, 38–42, 44–46], multicentric hospital-based in one study [36] and population-based in three studies [37, 43, 47]. In ten studies, survivors were diagnosed with PC, on average (mean, median), five to 10 years before the time of HRQoL assessment. [36–40, 43–47] In three studies, survivors were diagnosed more than 10 years before the time of HRQoL assessment. [35, 41, 42] Most studies included long-term PC survivors with localized (TNM stage: T1 & T2) and locally advanced (TNM stage: T3 & T4) PC [35, 38, 39, 42, 43, 45, 46] (categorization based on [48, 49]). Whereas two studies specifically recruited survivors after locally advanced PC [36, 44], four studies recruited survivors after only localized PC. [37, 39, 41, 47]. Ten studies [35–41, 45–47] provided no information on how they analyzed recurrent PC cancer survivors and whether recurrent PC cases were included in their dataset. Two studies [42, 43] included recurrent cancer patients and one excluded them, as they died during the follow-up time [44].

The average age of PC survivors at HRQoL assessment was around 75 years, ranging from 53 to 90 years of age. The RCTs, and some prospective cohort studies, only reported age at study enrollment (baseline). Thus, for these studies, the study population age at different HRQoL assessment time points can only be estimated.

One study excluded survivors with comorbid conditions [35], whereas four [43–45, 47] studies explicitly considered long-term PC survivors with comorbidities. These studies showed that >60% of long-term PC survivors were diagnosed with at least one comorbid condition.

#### Participation rate and number of participants

Sample size was defined at time of HRQoL assessment. Half of the studies had a sample size below 100 participants [35, 38, 39, 42, 45, 46], five had a sample size between 101 and 200 participants [36, 40, 41, 44, 47], one had 780 participants [43] and one study cohort consisted of 1463 participants 5 years post-randomization, with 1413 participants remaining for analysis 6 years post-randomization [37]. Participation rate (defined as the number of participants divided by the number of eligible patients at the time of long-term HRQoL assessment) was over 90% in one study [46], between 70 and 90% in ten studies [35–37, 39–45] and below 60% in one study [47].

#### Intervention comparisons and stage at diagnosis

Interventions were generally classified as RP, EBRT (referring to the external delivery of any type of radiation), ADT, BT, WW or AS. Studies either compared HRQoL by primary intervention in long-term survivors with:

1. localized PC only [37, 41, 47],
2. locally advanced PC only, [36, 44] or
3. localized or locally advanced PC [35, 38, 39, 42, 43, 45, 46] (Tables 4 and 5).

Unfortunately, one study did not reveal information about the cancer stage. This study was categorized as stage X [40] (Tables 4 and 5).

Additionally, alternative comparison methods for HRQoL among primary intervention groups were identified. Studies either compared:

1. HRQoL of PC survivors undergoing a specific primary intervention with controls from the general population at certain points over time [35, 38, 42, 45, 47],
2. HRQoL of PC survivors undergoing different interventions to each other at certain time points [36–41, 43, 46, 47] or.
3. HRQoL of PC survivors undergoing different interventions over a certain time period [36, 37, 44] (Tables 1 and 3).

**Table 3:** Instruments

	Instrument	Abbreviation	Frequency
Instruments to assess HRQoL	European Organisation for Research and Treatment of Cancer Core Questionnaire (30-items)	EORTC QLQ-C30	3
	36-item Short Form Health Survey	SF-36	7
	12-item Short Form Health Survey & European Organisation for Research and Treatment of Cancer Core Questionnaire (30-items)	SF-12 & EORTC QLQ-C30	1
	European Organisation for Research and Treatment of Cancer Core Questionnaire (30-items) & Quality of Life-Cancer Survivors questionnaire	EORTC QLQ-C30 & QoL-CS	2
Instruments to assess PC symptoms	Brief Male Sexual Inventory & University of California Los Angeles Prostate Cancer Index	BFSI & UCLA-PCI	1
	European Organisation for Research and Treatment of Cancer Prostate Cancer Specific Module (19 -items, pre-version of PR25)	EORTC QLQ-PR25/PR-19	1
	Expanded Prostate Cancer Index & International Continence Society Male Short-Form questionnaire & Hospital Anxiety and Depression Scale	EPIC & ICSaleSF & ICIQ & HADs	1
	Southwest Oncology Group Treatment Specific Measure	‚---‘	1
	Prostate Cancer Symptom Scale	PCSS	2
	International Prostate Symptom Score & International Index of Erectile Function	IPSS & IIEF	1
	University of California Los Angeles Prostate Cancer Index	UCLA-PCI	2
	International Prostate Symptom Score & University of California Los Angeles Prostate Cancer Index	IPSS & UCLA-PCI	1
	University of California Los Angeles Prostate Cancer Index & Hospital Anxiety and Depression Scale	UCLA-PCI & HADS	1
	Expanded Prostate Cancer Index & Dutch Sexual Activities Module	EPIC & SAc	1

Overall, EBRT was the most commonly evaluated intervention, followed by RP. The most common control group was the general population (*n* = 10) [35, 38, 42–44, 47] (Table 2).

### Assessment of health-related quality of life and prostate cancer specific symptoms

Included studies employed generic, as well as, disease-specific HRQoL instruments.(Table 3) Seven studies employed the SF-36 questionnaire as a generic HRQoL assessment instrument [40, 42–47], and five studies used the EORTC QLQ-C30 (Version 1.0 and 3.0) [35, 36, 38, 39, 41].

One study [37] used both the abbreviated form of the SF-36, the SF-12, and the EORTC QLQ C30. Additionally, two studies [43, 47] made use of the Dutch version of the Quality of Life-Cancer Survivors (QoL-CS) questionnaire [50]. The EORTC QLQ-C30 consists of five functional scales, nine symptom specific subscales and a global health status scale [25]. In contrast, both the SF-36 and the SF-12 consist of eight scales. The scales include general health perception, which encompasses two general domains: physical and mental well-being [26, 51]. Scales in both instruments are linearly transformed to values from 0 to 100 [52]. In the EORTC QLQ-C30, a high score for a functional scale represents a high/healthy level of functioning, a high score for the global health status/QoL represents a high QoL. Generally, a high score for a symptom scale/item represents a high level of symptomatology [52]. Most studies reported statistically significant differences [36–41, 43–47]. Five studies completed an additional analysis if the results were clinically meaningful [35–37, 39, 43].

PC specific symptoms were assessed with 11 different instruments. [53–63] (Table 3). Additionally, the Hospital Anxiety and Depression Scale (HADS) [64] was used in two studies. [37, 46] Six studies [37, 38, 40, 41, 45, 46] combined different instruments, six [35, 36, 39, 42, 44, 47] used one instrument, and one study did not assess PC specific symptoms [43]. Scales of disease-specific HRQoL instruments were mainly related to urinary, bowel and sexual functions/problems.

### Study findings

Overall, studies were heterogeneous and most had potential limitations. Therefore, we decided to systematically report but not pool (e.g. in a meta-analysis), the main results. Further, we divided the results between RCTs and observational studies and grouped them by disease stage. (Tables 4 and 5, Additional file 1: Appendix Tables B and C).

**Table 4:** Main findings on HRQoL in RCTs

Comp.:	Study	Key Findings	Potential Limitation(s)
S1^a^	Donovan, J L/ 2016 [37]	Comparison: AS vs. RP vs. EBRT, follow-up time^b^: 5-6 years, mean age^c^: 62 years - No significant differences were observed among intervention groups in measures of general health-related or cancer-related quality of life	
S1	Giberti, C/ 2009 [41]	Comparison: RP vs. BT, follow-up time^b^: 5 years, mean age^c^: 65.3 years - No significant differences were observed among intervention groups in measures of general health-related or cancer-related quality of life	- Sample size <100 in both study arms - No intention to treat analyses
S2	Brundage, M/ 2015 [36]	Comparison: ADT vs. ADT + EBRT, follow-up time^b^: 5-8 years, median age^c^: 69.7 years - No significant between-arm differences in physical or role functioning at any time point 5+ years after diagnosis - Significant (*p* < 0.001) deterioration in both arms over time for physical and role functioning	- Sample size <100 in both study arms - Only results on physical and role functioning were reported for this follow-up time

*Comp.* Comparison group

S1: HRQoL by primary intervention in long-term survivors with localized PC; S2: HRQoL by intervention in long-term survivors with locally advanced PC; S3: HRQoL by intervention in long-term survivors with localized or locally advanced PC

Studies were ordered by stage information and within each group alphabetically

As potential limitation following criteria were considered: (1) sample size 100 per study arm for studies using EORTC-C30 and 70 for studies using SF-36 (2) randomization (3) intention to-treat analyses (4) reporting of results appropriate

^a^Inlcusion of PC survivors with disease progression

^b^Time since randomization

^**c**^Age at randomization

**Table 5:** Main findings on HRQoL in observational studies

Comp.	Study	Key Findings	Potential Limitation(s)
S1^a^	Thong, M S/ 2010 [47]	Comparison: AS vs. EBRT, follow-up time^b^: 7.8 years, mean age^d^: 75.8 years - No significant differences in HRQL between AS and RT on the QOL-CS scales - In multivariate models RT was significantly negatively associated with physical functioning, bodily pain dimensions, QOL-CS spiritual and total well-being scores Subgroup analyses: exclusion of clinically progressed cancer survivors - Above results remain unchanged Comparison: AS or EBRT vs. controls from the general population, follow-up time^b^: 7.8 years, mean age^d^: 75.8 years - PC survivors reported comparable HRQL scores compared to an age-matched, normative population, except in role physical PC survivors treated with EBRT reported significantly (*p* < 0.05) worse mean compared to controls from the general population	- No baseline data available
S2	Namiki, S/ 2011 [44]	Comparison: RP vs. EBRT, follow-up time^b^: 5 years, mean^e^: 69.5 years - Patterns of alterations over time in intervention groups were different in physical function (*p* < 0.001), role physical (*p* < 0.001), role emotional (*p* < 0.001) and vitality (*p* = 0.027), whereas survivors treated with RP had higher scores in all domains	- Sample size <70 in all study arms - (Repeated ANOVA-tests: only changes over time are shown) - No confounding control - No adjustment for attrition error
S3^a^	Berg, A/ 2007 [35]	Comparison: EBRT + ADT/clinical progression vs. controls from the general population, follow-up time^b^: 10-16 years, median age^e^: 66 years - Worse clinically relevant scores for survivors in social functioning scales and higher burden with insomnia and diarrhea Comparison: EBRT vs. controls from the general population, follow-up time^b^: 10-16 years, median age^e^: 66 years - Clinically relevant higher burden for PC survivors with diarrhea	- Sample size <100 in all study arms - No confounding control - No significance statistical test -No adjustment for attrition error
S3^a^	Fransson, P/ 2008 [38]	Comparison: EBRT vs. controls from the general population, follow-up time^c^: 15 years, mean age^d^: 78.1 years - No significant differences were observed among intervention groups in measures of general health-related or cancer-related QoL	- Sample size <100 in study arms - No confounding control - No adjustment for attrition error
S3	Fransson, P/ 2009 [39]	Comparison: EBRT vs. WW, follow-up time^c^: 10 years,median age^d^: 78 years - No significant differences were observed between groups in measures of general health-related or cancer-related QoL	- Sample size <100 in both study arms
S3	Johnstone, P A S/ 2000 [42]	Comparison: EBRT (plus ADT) vs. controls from the general population, follow-up time^c^: 13.9 years, median age^d^: 80 years - Clinically important differences^f^ but worse score for PC survivors in role emotional and vitality not statistically relevant	- Sample size <100 in both study arms - Only results on physical and role functioning were reported for this follow-up time
S3	Mols, F/ 2006 [43]	Comparison: RP vs. EBRT (plus ADT) vs. ADT vs. WW, follow-up time^b^: 5-10 years, age^d^: average 80 years - PC survivors who underwent RP had, in general, the highest HRQoL, followed by survivors who received WW and patients who received EBRT. Survivors who received ADT had the lowest physical HRQL, in general. - Significantly different means between intervention groups in physical functioning (*p* < 0.001, clinical important difference^f^) and physical well-being (*p* = 0.02). Clinically important differences^f^ in vitality among group means, but not significantly different means. - PC survivors treated with EBRT reported a significantly (*p* < 0.05) worse mean in physical functioning compared to survivors treated with EBRT - Survivors treated with ADT reported a significantly (*p* < 0.05) worse mean in physical functioning and vitality compared to survivors treated with EBRT Subgroup analyses – age groups: <75 years vs. > = 75 years - In general, HRQL scores were higher for younger survivors than for older survivors Comparison: RP or EBRT or ADT or WW vs. general population, 5-10 years after diagnosis - PC survivors reported comparable HRQL scores compared to an age-matched, normative population group - PC survivors treated with RP, EBRT and WW reported less problems with bodily pain than population controls	- Sample size <100 in both study arms - No intention to treat analyses
S3	Namiki, S/ 2014 [45]	Comparison: RP vs. controls from the general population, follow-up time^c^: 8.3 years, mean age^d^: 63.9 years - No significant differences were observed among the groups in measures of general health-related or cancer-related quality of life	- Sample size <70 in study arms - No adjustment for attrition error
S3^a^	Shinohara, N/ 2013 [46]	Comparison: EBRT vs. RP, localized and locally advanced PC, follow-up time: 5 years, mean/median age: 68 years - No significant differences were observed among the groups in measures of general health-related or cancer-related QoL	- Sample size <70 in all study arms - No adjustment for attrition error - No confounding control
X	Galbraith, M E/ 2005 [30]	Comparison: EBRT – LD^g^, EBRT – C^g^ vs. WW, follow-up time^c^: 5.5 years, age^d^: average 69.7 years - Regardless of type of intervention, health-related QOL and general health tend to decrease for prostate cancer survivors - PC survivors in WW tended to have poorer health outcomes	- Sample size <70 in all study arms - No confounding control - For growth curve analyses plots are printed badly, so it cannot be distinguished between intervention arms - For comparisons at specific time points it is not explained which statistical tests was used - *P*-values are not shown for all comparisons, not explained for which reasons some results are not shown - No adjustment for attrition error

*Comp.* Comparison group

S1: HRQoL by primary intervention in long-term survivors with localized PC; S2: HRQoL by intervention in long-term survivors with locally advanced PC; S3: HRQoL by intervention in long-term survivors with localized or locally advanced PC; X: No assignment possible as study revealed no information about cancer stage

Studies were ordered by stage information and within each group alphabetically

As potential limitations, the following criteria were considered: (1) sample size 100 per study arm for studies using EORTC-C30 and 70 for studies using SF-36 70 (2) adjustment for attrition error (3) statistical significance tests performed (4) adjustment for attrition error (only prospective cohort studies) (5) baseline data available (6) reporting of results appropriate

Definition of clinically meaningful difference: EORTC QLQ-C30: min. 10 points difference; SF-36: min. 5 points difference in general health dimension, min 6.5 points in physical dimension, 7.9 points in mental health dimension

^a^Inlcusion of PC survivors with disease progression

^b^Time since diagnosis

^c^Time since enrolment in study

^d^Age at survey

^e^Age at enrollment in study

^f^Not reported, but clinically meaningful difference

^g^EBRT-LD — Low-dose mixed-beam radiation, EBRT-C — Conventional radiation

#### HRQoL by primary intervention in long-term survivors with localized PC

Three studies assessed HRQoL in long-term survivors with localized stage PC [37, 41, 47]. Comparisons were drawn from two RCTs, comparing either AS vs. RP vs. EBRT, or RP vs. EBRT [37, 41] and one observational study comparing AS vs. EBRT. Both interventions used controls from the general population [47].

These three studies showed that long-term survivors with localized stage PC have comparable HRQoL independent from the chosen intervention. (Tables 4 and 5) Moreover, one study revealed that PC survivors do not experience any reduction in their HRQoL, except for deficits in physical function, when compared with controls from the general population. [47] However, in two studies [37, 47] EBRT had an effect on bowel function. Additionally, one RCT reported that RP had the greatest negative effect on urinary and sexual function, compared to survivors on AS or survivors treated with EBRT [37] (Additional file 1: Appendix Tables B and C).

#### HRQoL by primary intervention in long-term survivors with locally advanced PC

Two studies (one RCT, one observational study) assessed HRQoL in long-term survivors with locally advanced PC [36, 44]. The RCT compared PC survivors treated with ADT vs. ADT + EBRT [36] and the observational study RP vs. EBRT [44]. Only the RCT reported results for intervention comparisons at specific time points. In this RCT, no difference in HRQoL or PC symptoms could be identified. After 5 years, the observational study shows both interventions have good outcomes, whereas PC patients treated with RP reported better well-being [36].

#### HRQoL by primary intervention in long-term survivors with localized or locally advanced PC

Seven observational studies compared HRQoL in survivors with localized and locally advanced stage PC [35, 38, 39, 42, 43, 45, 46]. In four studies [35, 38, 42, 43], PC survivors treated with EBRT were compared with controls from the general population, whereas in three [35, 42, 43] of these four studies, PC survivors were additionally treated with ADT. In these four studies, no uniform pattern in HRQoL differences could be identified. Three [35, 38, 42] studies reported significant, or even clinically relevant, functioning in different HRQoL domains (social, role and emotional functioning) and a higher burden of diarrhea, appetite loss, nausea, pain and insomnia. Conversely, the fourth study [43] revealed that patients reported comparable HRQoL, and less bodily pain, in comparison to a control group from the general population. However, for PC specific symptoms, authors could identify more detriments in sexual function domains (*n* = 2) [35, 42] and more urinary bowel problems (*n* = 2) [38, 42] when compared to controls from the general population (Tables 4 and 5, Additional file 1: Appendix Tables B and C).

When PC survivors treated with EBRT were compared to either PC survivors treated with RP or WW, no significant results in HRQoL could be identified [39, 46]. The same result applies for the comparison of PC survivors treated with RP vs. controls from the general population [45].

The one study comparing PC survivors treated with RP vs. EBRT vs. ADT vs. WW showed significant differences were observed in physical functioning and physical well-being, whereas survivors treated with RP had the best scores in these domains. Further, survivors treated with ADT had the lowest scores. In a separate analysis comparing all the intervention groups with controls from the general population, no intervention group reported worse HRQoL [43].

## Discussion

Five and 10 year PC-specific survival rates are nearing 100%, seemingly independent from type of primary intervention [18]. Consequently, experts continue to disagree on a preferred intervention course, particularly in the disease’s early stages.

This review identified 13 studies (three RCTs and 10 observational studies), which evaluated HRQoL and PC specific symptoms in long-term PC survivors at different cancer stages. Studies varied in terms of intervention comparison groups, instruments used, and whether/how studies reported results on primary interventions for localized PC, locally advanced PC, or on both together without distinction.

The main tested intervention group was EBRT (plus ADT), and only limited information was available on PC survivors treated with ADT only, and on PC survivors on AS or WW. AS and WW are only recently considered standard care. Thus, the lack of studies in this review focusing on long-term PC survivors (and two earlier reviews including short-term survivors) undergoing AS or WW, is not surprising [65, 66]. The limited number of studies assessing HRQoL in PC survivors treated with ADT is also logical, as ADT is mainly indicated in patients with advanced stage PC, which has a shorter survival time [67].

To assess generic HRQoL, studies either used the SF-36, or EORTC QLQ-C30, thus allowing for comparisons to be drawn across at least some domains. However, our review reveals a diverse number of instruments employed in assessing PC specific symptoms. UCLA-PCI (*n* = 4) was the most commonly employed instrument, followed by the EPIC (*n* = 2) and IPSS (*n* = 2). The first two questionnaires (UCLA-PCI and EPIC) focus on urinary, sexual and bowel symptoms, whereas the latter (IPSS) evaluates only urinary symptoms. The studies in this review: (1) focused on only one questionnaire, (2) used different combinations of the questionnaires, or (3) did not evaluate PC specific symptoms at all, making it impossible to pool results across studies.

Interestingly, the RCTs evaluated in this systematic review included either PC survivors with localized PC [37, 41] or locally advanced PC [36], whereas only two observational studies [44, 47] made this distinction. Therefore, the results of these observational studies should be interpreted carefully, because the choice of intervention is dependent on stage at diagnosis [10].

In addition to the use of diverse instruments, the majority of reviewed studies had potential limitations. These limitations prevented our ability to draw firm conclusions on HRQoL’s dependency on primary intervention in long-term PC survivors. First, only three studies [37, 43, 47] had sufficient power to detect predetermined differences in scores between groups. For example, to detect a difference of ten points with a power of 80% and alpha = 0.05, a sample size of 100 per group in the EORTC QLQ-C30, and of around 70 in the SF-36 questionnaire, is needed. [68, 69] Second, ten studies [31, 32, 34, 35, 38–43] were prone to confounding, as they were observational studies. In these observational studies, control for potential confounding was performed to varying degrees by only half of the studies [31, 34, 39, 42, 43]. Age, stage, comorbidity and other factors are strongly associated with HRQoL and with intervention decision. Thus, observational studies should carefully account for potential confounding by these factors. Third, most studies did not assess the results’ clinical significance [34, 36–38, 40–43], which limits clinical relevance. Finally, selection bias may occur if patients experiencing PC recurrence are excluded from sample analysis. Only two studies explicitly stated whether survivors with recurrent disease were included in the analysis, or not.

The strong heterogeneity across studies, and their potential limitations, reveals an urgent need for more high-quality, large-scale, prospective cohort studies, or RCTs with repeated follow-up HRQoL assessments.

However, some robust data exist from two RCTs and one population-based observational, retrospective cohort study comparing HRQoL by primary intervention in survivors with localized stage PC. The data do not suggest HRQoL differs by intervention. However, these three studies had different comparisons and included, in total, four different interventions, whereas pooling of study findings was not possible.

No consistent results could be seen in other studies based on survivors with locally advanced PC, or on combining localized or locally advanced PC stage. Intervention detriments are seen for various scales: (1) physical well-being, (2) social and role function, (3) vitality and (4) role emotional. However, results are contractionary due to the previously discussed limitations and the heterogeneity of included studies. Therefore, the question of whether HRQoL varies by primary intervention and (if yes), which intervention options are superior with respect to HRQoL, cannot be answered based on these studies.

Further, our systematic review has some of its own limitations. As the aim was to compare the influence of primary interventions on HRQoL in long-term PC survivors, all studies that did not have a comparison group (either general population or another intervention group) were excluded from the review. Additionally, qualitative studies were not included as we only wanted to review and compare quantitative studies using validated questionnaires. Furthermore, as consensus exists that HRQoL is a multidimensional concept that encompasses all aspects of survivors’ well-being, three studies that reported or assessed HRQoL on only one domain were not included. Additionally, due to the limitations and variations of the instruments, and comparison groups of the included studies, result pooling was not possible for the observational studies, or for the RCTs.

## Conclusion

Despite an increasing number of publications studying HRQoL and/or disease specific symptoms in PC survivors, only a limited number of publications is available focusing on long-term PC survivors and primary intervention. This systematic review exposes the heterogeneity of PC intervention studies in terms of (1) stage at diagnosis, (2) intervention groups and (3) instruments used. In addition, most studies are limited by low sample size, and in the case of observational studies, potential confounding by indication, or due to insufficient adjustment.

Robust data from two RCTs and one observational study, comparing HRQoL by primary intervention in localized PC survivors, suggest that HRQoL does not seem to differ by intervention. However, data from observational studies assessing HRQoL by primary intervention of PC survivors and combining localized, or locally advanced stage PC, identified differences for various scales: physical well-being, social and role function, vitality and role emotional. However, study heterogeneity and limitations prevent the identification of clear patterns.

Therefore, a review of the existing studies reveals an urgent need for more high-quality, large-scale, prospective cohorts or RCTs with repeated follow-up HRQoL assessments in order to provide clinicians and patients with sound evidence. Currently, it is unclear whether HRQoL varies by primary intervention and (if yes) which primary intervention is superior with respect to long-term HRQoL in PC patients. Additionally, studies should indicate clinical meaningfulness in addition to statistically significant differences, in order to better inform patient/caregiver decision-making.

Additionally, when HRQoL is assessed, domains other than physical well-being and PC specific problems (e.g. incontinence or impotence) should be addressed, as differences occurred in various scales.

## Additional file

Additional file 1: Appendices. (DOCX 41 kb)

## Abbreviations

ADT: Androgen deprivation therapy
AS: Active surveillance
BT: Brachytherapy
CK: Cyberknife
CRYO: Cryotherapy
CT: Chemotherapy
EBRT: External beam radiation therapy
HRQoL: Health-related quality of life
PC: Prostate cancer
QoL: Quality of life
RCT: Randomized controlled trial
RP: Radical prostatectomy
WW: Watchful waiting
