# Correction to: Health-related quality of life among long-term (≥5 years) prostate cancer survivors by primary intervention: a systematic review

**DOI:** 10.1186/s12955-018-0968-x

**Published:** 2018-08-07

**Authors:** Salome Adam, Anita Feller, Sabine Rohrmann, Volker Arndt

**Affiliations:** 1 Division of Chronic Disease Epidemiology, Epidemiology, Biostatistics and Prevention Institute, University of Zurich, Zurich, Switzerland; 2 Unit of Cancer Survivorship, Division of Clinical Epidemiology and Aging Research, German Cancer Research Center (DKFZ), Heidelberg, Germany; 3 National Institute for Cancer Epidemiology and Registration (NICER), University of Zurich, Zurich, Switzerland; 4 National Institute for Cancer Epidemiology and Registration (NICER), University of Zurich, Zurich, Switzerland; 5 Unit of Cancer Survivorship, Division of Clinical Epidemiology and Aging Research, German Cancer Research Center (DKFZ), Heidelberg, Germany

## Correction

The original article [1] contains errors whereby some information provided in Tables 2 and 5 in the online version is missing in the PDF version; in addition, some details regarding the study by Mols et al., Johnstone et al. and Fransson et al. (2008) in Tables 1 and 5 require correction.

**Table 1:** Characteristics of included studies

		At survey ≥ 5 years		Mean/ Median (Range)^a^		At diagnosis^g^
First Author/ Year, Country	Study Design	Sample Size (n)	Intervention (%)	Age at survey (years)	Follow-up time^f^ (years)	Cancer Stage (%)
Berg, A/ 2007, Norway [35]	Hospital-based observational prospective monocentric cohort study	64	EBRT (100) [+ADT (44.0)]^e^	66^c^ (48-81)	11 (10 – 16)	Localized PC (33.0) Locally advanced PC (67.0)
Brundage, M/ 2015, UK and US [36]	Hospital-based mulitcentric randomized controlled trial	85-111^d^	1. ADT (50.0)^c^ 2. ADT + EBRT (50.0)^c^	69.7^c^ (65.5 – 73.5)	(5 – 8)	Locally advanced PC (100.0)
Donovan, J L / 2016, UK [37]	Population-based multicentric randomized controlled trial	1413-1463^d^	1. AS (33.2) 2. RP (33.7) 3. EBRT (33.1)	62^c^	(5 – 6)	Localized PC (100.0)
Fransson, P/ 2008, Sweden [38]	Hospital-based observational prospective monocentric cohort study	64	1. EBRT (42.2) +ADT (20.3) 2. Controls (57.8)	78.1 (62 – 87)	14.7 (13.5 – 16.4)	Localized PC (89.9) Locally advanced PC (11.1)
Fransson, P/ 2009, Sweden [39]	Hospital-based observational monocentric retrospective cohort study	54	1. EBRT (50.0) 2. WW (50.0)	78 (54 – 88)	9.6 (6.4 – 16.3)	Local PC (100.0)
Galbraith, M E/ 2005, US [40]	Hospital-based observational prospective monocentric cohort study	137	1. WW (11.5)^c^ 2. RP (21.4)^c^ 3. EBRT – C (9.9)^b,c^ 4. EBRT - PB (11.5)^b,c^ 5. EBRT - MB (20.3) ^b,c^ 6. EBRT -LD (13.7)^b,c^ 7. EBRT - HD (17)^b,c^	69.9^c^	5.5	No information
Giberti, C/ 2009, Italy [41]	Hospital-based monocentric randomized controlled trial	174	1. RP (44.5) 2. BT (55.5)	65.3^c^ (56 – 74)^c^	5	Localized PC (100.0)
Johnstone, P A S/ 2000, US [42]	Hospital based observational monocentric prospective cohort study	46	EBRT (100.0) [+ ADT (43.5)]^4^	80 (62 – 90)	13.9 (10 – 23)	Localized PC Locally advanced PC
Mols, F/ 2006, Denmark [43]	Population-based observational retrospective cohort study	780	1. RP (32.9) 2. EBRT (41.4) 3. ADT (13.7) 4. WW (11.9)	75	(5-10)	Localized PC (76.0) Locally Advanced PC (18.0) Unknown (6.0)
Namiki, S/ 2011, Japan [44]	Hospital-based observational prospective monocentric cohort study	111	1. RP (43.2) + ADT (48) 2. EBRT (56.8) + ADT (100.0)	69.5^c^ (53 – 84)	5	Locally Advanced PC (100.0)
Namiki, S/ 2014, Japan [45]	Hospital-based observational prospective monocentric cohort study	91	RP (100.0)	63.9^c^	8.5 (7.1 – 10.25)	Localized PC (94.5) Locally Advanced PC (5.5)
Shinohara, N/ 2013, Japan [46]	Hospital-based observational monocentric prospective cohort study	67	1. EBRT (32.4) 2. RP (67.6)	68^2^ (53 – 79)	5	Localized PC (93.4) Locally Advanced PC (6.6)
Thong, M S/ 2010, Netherlands [47]	Population-based observational retrospective cohort study	142	1. AS (50.0) [+ ADT (2.8)/ +RP (1.4)/ + EBRT (7)/ + EBRT + ADT (1.4)]^e^ 2. EBRT (50) + [RP (7)/ + ADT (2.8)/ +EBRT (1.4) + EBRT + ADT (1.4)]^e^	75.8	7.8	Localized PC (100)

*RP* Radical Prostatectomy, *EBRT* External Beam Radiotherapy (refers to the external delivery of any type of radiation), *BT* Brachytherapy, *WW* Watchful Waiting, *AS* Active Surveillance, *ADT* Androgen Deprivation Therapy

^a^Mean/Medians for total sample

^b^EBRT-C — Conventional radiation; EBRT-HD — High-dose mixed-beam radiation; EBRT-LD — Low-dose mixed-beam radiation; EBRT-MB — Standard protocol/mixed-beam radiation; EBRT-PB — Proton beam radiation

^c^Sample size/Age at enrolment in study or randomisation

^d^Sample sizes at different time points ≥ 5 years

^e^Secondary intervention(s)

^f^Either time since diagnosis or time since randomization

^g^Categorization: local PC – T1 & T2, locally advanced PC T3 & T4

**Table 2:** Summary table of study characteristics

Characteristic						Frequency
Study Design	Randomized controlled trial Observational prospective cohort study Observational retrospective cohort study					3 7 3
Recruitment	Monocentric hospital-based Multicentral hospital-based Population-based					9 1 3
Comparison: Intervention vs. general population*	RP	EBRT	ADT	WW	AS	
	X					2
		X^1a^				5
			X			1
				X		1
					X	1
Comparison between different interventions*	RP	EBRT	ADT	WW	AS	
	X	X			X	1
	X	X^d^				1
	X	X				1
		X vs. X^c^				1
		X^c^	X			1
		X			X	1
		X		X		1
	X	X	X	X		1
	X	X^e^		X		1
	X	X^f^				1
Sample sizes (total population)	<100 101 – 200 780 1463 (after 5 years since randomization) respectively 1413 participants (6 years since randomization)					6 5 1 1
Years since diagnosis/randomization	Long-term survivors (5-10 years after diagnosis) Very long-term survivors (10 + years after diagnosis)					10 3
Stage at diagnosis	Localized (T1/T2) PC Locally advanced (T3/T4 any N1/M1) PC Localized & locally advanced PC No information					3 2 7 1
Recurrent PC survivors	No information Excluded Included					10 1^g^ 2
Progressive PC survivors	No information Excluded Included					5 3 5

^a^Some studies had multiple comparisons

^b^“Plus ADT and/or clinical progression”

^c^plus ADT

^d^Brachytherapy

^e^EBRT-C — Conventional radiation; EBRT-HD — High-dose mixed-beam radiation; EBRT-LD — Low-dose mixed-beam radiation; EBRT-MB — Standard protocol/mixed-beam radiation; EBRT-PB — Proton beam radiation

^f^Brachytherapy

^g^Excluded because they died

**Table 5:** Main findings on HRQoL in observational studies

Comp.	Study	Key Findings	Potential Limitation(s)
S1^a^	Thong, M S/ 2010 [47]	Comparison: AS vs. EBRT, follow-up time^b^: 7.8 years, mean age^d^: 75.8 years - No significant differences in HRQoL between AS and EBRT on the QOL-CS scales - In multivariate models EBRT was significantly negatively associated with physical functioning, bodily pain dimensions, QOL-CS spiritual and total well-being scores Subgroup analyses: exclusion of clinically progressed cancer survivors - Above results remain unchanged Comparison: AS or EBRT vs. controls from the general population, follow-up time^b^: 7.8 years, mean age^d^: 75.8 years - PC survivors reported comparable HRQoL scores compared to age-matched, normative population, except in role physical PC survivors treated with EBRT reported significantly (p<0.05) worse mean compared to controls from the general population	- No baseline data available
S2	Namiki, S/ 2011 [44]	Comparison: RP vs. EBRT, follow-up time^b^: 5 years, mean^e^: 69.5 years - Patterns of alterations over time in intervention groups were different in physical function (p<0.001), role physical (p<0.001), role emotional (p<0.001) and vitality (p=0.027), whereas survivors treated with RP had higher scores in all domains	- Sample size <70 in all study arms - (Repeated ANOVA-tests: only changes over time are shown) - No confounding control - No adjustment for attrition error
S3^a^	Berg, A/ 2007 [35]	Comparison: EBRT + ADT/clinical progression vs. controls from the general population, follow-up time^b^: 10-16 years, median age^e^: 66 years - Worse clinically relevant scores for survivors in social functioning scales and higher burden with insomnia and diarrhea Comparison: EBRT vs. controls from the general population, follow-up time^c^: 10-16 years, median age^e^: 66 years - Clinically relevant higher burden for PC survivors with diarrhea	- Sample size <100 in all study arms - No confounding control - No significance statistical test -No adjustment for attrition error
S3^a^	Fransson, P/ 2008 [38]	Comparison: EBRT vs. controls from the general population, follow-up time^c^: 15 years, mean age^d^: 78.1 years - Significantly different (p<0.05) worse mean for PC survivor in role function (clinically important difference)^f^ and higher burden with appetite loss, diarrhea (clinically important difference)^f^, nausea/vomiting and pain Comparison: EBRT vs. EBRT + ADT, follow-up time^c^: 15 years, mean age^d^: 78.1 years - No significant differences were observed among intervention groups in measures of general health-related or cancer-related QoL	- Sample size <100 in study arms - No confounding control - No adjustment for attrition error
S3	Fransson, P/ 2009 [39]	Comparison: EBRT vs. WW, follow-up time^c^: 10 years, median age^d^: 78 years - No significant differences were observed between groups in measures of general health-related or cancer-related QoL	- Sample size <100 in both study arms
S3	Johnstone, P A S/ 2000 [42]	Comparison: EBRT (plus ADT) vs. controls from the general population, follow-up time^c^: 13.9 years, median age^d^: 80 years - Clinically important differences^f^ but worse scores for PC survivors in role emotional and vitality not statistically relevant	- Sample size <70 in study arm - No statistical significance test performed - No confounding control - No baseline data
S3	Mols, F/ 2006 [43]	Comparison: RP vs. EBRT (plus ADT) vs. ADT vs. WW, follow-up time^b^: 5-10 years, age^d^: average 80 years - PC survivors who underwent RP had, in general, the highest HRQoL, followed by survivors who received WW and patients who received EBRT. Survivors who received ADT had the lowest physical HRQL, in general. - Significantly different means between intervention groups in physical functioning (p < 0.001, clinical important difference^f^) and physical well-being (p = 0.02). Clinically important differences^f^ in vitality among group means, but not significantly different means. - PC survivors treated with EBRT reported a significantly (p < 0.05) worse mean in physical functioning compared to survivors treated with RP - Survivors treated with ADT reported a significantly (p<0.05) worse mean in physical functioning and vitality compared to survivors treated with RP Subgroup analyses – age groups: <75 years vs. ≥75 years - In general, HRQoL scores were higher for younger survivors than for older survivors Comparison: RP or EBRT or ADT or WW vs. general population, 5-10 years after diagnosis - PC survivors reported comparable HRQoL scores compared to an age-matched, normative population group - PC survivors treated with RP, EBRT and WW reported less problems with bodily pain than population controls	- Sample size <70 in two (ADT & WW) out of 4 study arms in general analyses - Sample size <70 in three out of 4 study arms (RP, ADT & WW) in subgroup analyses - No baseline data available
S3	Namiki, S/ 2014 [45]	Comparison: RP vs. controls from the general population, follow-up time^c^: 8.3 years, mean age^d^: 63.9 years - No significant differences were observed among the groups in measures of general health-related or cancer-related quality of life	- Sample size <70 in study arms - No adjustment for attrition error
S3^a^	Shinohara, N/ 2013 [46]	Comparison: EBRT vs. RP, localized and locally advanced PC, follow-up time: 5 years, mean/median age: 68 years - No significant differences were observed among the groups in measures of general health-related or cancer-related QoL	- Sample size <70 in all study arms - No adjustment for attrition error - No confounding control
X	Galbraith, M E/ 2005 [30]	Comparison: EBRT – LD^g^, EBRT – C^g^ vs. WW, follow-up time^c^: 5.5 years, age^d^: average 69.7 years - Regardless of type of intervention, health-related QOL and general health tend to decrease for prostate cancer survivors - PC survivors in WW tended to have poorer health outcomes	- Sample size <70 in all study arms - No confounding control - For growth curve analyses plots are printed badly, so it cannot be distinguished between intervention arms - For comparisons at specific time points it is not explained which statistical tests was used - P-values are not shown for all comparisons, not explained for which reasons some results are not shown - No adjustment for attrition error

Comp. Comparison group

S1: HRQoL by primary intervention in long-term survivors with localized PC; S2: HRQoL by intervention in long-term survivors with locally advanced PC; S3: HRQoL by intervention in long-term survivors with localized or locally advanced PC; X: No assignment possible as study revealed no information about cancer stage

Studies were ordered by stage information and within each group alphabetically.

As potential limitations, the following criteria were considered: (1) sample size 100 per study arm for studies using EORTC-C30 and 70 for studies using SF-36 70 (2) adjustment for attrition error (3) statistical significance tests performed (4) adjustment for attrition error (only prospective cohort studies) (5) baseline data available (6) reporting of appropriate results.

Definition of clinically meaningful difference: EORTC QLQ-C30: min. 10 points difference; SF-36: min. 5 points difference in general health dimension, min 6.5 points in physical dimension, 7.9 points in mental health dimension.

^a^Inlcusion of PC survivors with disease progression

^b^Time since diagnosis

^c^Time since enrolment in study

^d^Age at survey

^e^Age at enrollment in study

^f^Not reported, but clinically meaningful difference

^g^EBRT-LD — Low-dose mixed-beam radiation, EBRT-C — Conventional radiation

As such, the corrected tables can be seen ahead.
