# Supplementary material for: Health-related quality of life among long-term (≥5 years) prostate cancer survivors by primary intervention: a systematic review
Source: Health Qual Life Outcomes. 2018 Jan 23;16:22. doi: 10.1186/s12955-017-0836-0 (PMC5781262; doi:10.1186/s12955-017-0836-0)
Supplement: Additional file 1: — Appendices. (DOCX 41 kb) [file 12955_2017_836_MOESM1_ESM.docx]

**Appendices**

**A: Search strategy**

**Total 2054 (1230)**

**CINAHL 65 (17)**

TI ("quality of life" OR hrqol) OR ((MH "Quality of Life+") OR (MH "Quality-Adjusted Life Years") OR (MH "Patient Satisfaction") OR TI ("quality of life" OR hrqol OR "patient satisfaction" OR well-being) OR AB ("quality of life" OR hrqol OR "patient satisfaction" OR well-being)) AND ((MH "Questionnaires+") OR (MH "Scales") OR (MH "Outcome Assessment") OR (MH "Patient Assessment") OR TI (assess* OR evaluat* OR measure* OR determin*) OR AB (assess* OR evaluat* OR measure* OR determin*)) OR TI ("Prostate Cancer Index" OR EORTC OR QLQ-C30 OR EORTC OR PR-25 OR EPIC OR SF-36) OR TI ("Prostate Cancer Index" OR EORTC OR QLQ-C30 OR EORTC OR PR-25 OR EPIC OR SF-36) AND ((MH "Cancer Survivors") OR (MH "Survivors+") OR TI surviv* OR AB surviv*) AND (TI ('long term' OR longterm OR ((yr OR year*) N3 after) OR ((yr OR year*) N6 surviv*)) OR AB ('long term' OR longterm OR ((yr OR year*) N3 after) OR ((yr OR year*) N6 surviv*))) AND (MH "Prostatic Neoplasms+") OR TI ((prostate OR prostatic) N3 (cancer OR adenocarcinoma* OR carcinoma* OR neoplasia OR neoplasm*)) OR AB ((prostate OR prostatic) N3 (cancer OR adenocarcinoma* OR carcinoma* OR neoplasia OR neoplasm*))

**EMBASE 720 (589)**

'quality of life’:ti OR ' hrqol’ ti OR l 'quality of life'/exp OR 'patient satisfaction'/exp OR 'wellbeing'/exp OR 'quality of life':ab,ti OR hrqol:ab,ti OR 'patient satisfaction' ab,ti OR 'well being' ab,ti OR AND ('questionnaire'/exp OR 'rating scale' /exp OR 'scoring system' /exp OR 'disease assessment'/exp OR 'general health status assessment'/exp OR assess*:ab,ti ORevaluat*:ab,ti OR measure*:ab,ti OR determin*:ab,ti) OR 'prostate cancer index':ab,ti OR 'qlq c30':ab,ti OR eortc:ab,ti OR 'pr 25':ab,ti epic 'sf 26:ab,ti AND 'survivor'/de OR 'cancer survivor'/exp OR surviv*:ab,ti AND ('long term':ab,ti OR longterm:ab,ti OR (( yr OR year*) NEAR/3 after): ab,ti OR ((yr OR year*) NEAR/6 surviv*):ab,ti) AND ‘prostate cancer?exp OR ((prostate OR prostatic) NEAR/3 (cancer OR adenocarcinoma* oR canrcinoma* OR neoplasia OR neoplasm*)):ab, ti

**Medline 361 (94)**

TI ("quality of life" OR hrqol) OR ((MH "Quality of Life") OR (MH "Quality-Adjusted Life Years") OR (MH "Patient Satisfaction+") OR TI ("quality of life" O hrqol OR "patient satisfaction" OR well being) OR AB ("quality of life" OR hrqol O "patient satisfaction" OR well-being)) AN ((MH "Surveys and Questionnaires+") O (MH "Outcome Assessment (Health Care)" OR (MH "Patient Outcome Assessment") OR TI (assess* OR evaluat* OR measure* OR determin*) OR AB (assess* OR evaluat* OR measure* OR determin*)) OR TI ("Prostate Cancer Index" OR EORTC OR QLQ-C30 OR EORTC OR PR-25 OR EPIC OR SF-36) OR TI ("Prostate Cance Index" OR EORTC OR QLQ-C30 O EORTC OR PR-25 OR EPIC OR SF-36) AND ((MH "Cancer Survivors") OR (MH "Survivors+") OR TI surviv* OR AB surviv*) AND (TI ('long term' OR longterm OR ((y OR year*) N3 after) OR ((yr OR year*) N6 surviv*)) OR AB ('long term' OR longterm OR ((yr OR year*) N3 after) OR ((yr OR year*) N6 surviv*))) AND (MH "Prostatic Neoplasms+") OR TI ((prostate OR prostatic) N3 (cancer OR adenocarcinoma* OR carcinoma* OR neoplasia OR neoplasm*)) OR AB ((prostate OR prostatic) N3 (cancer OR adenocarcinoma* OR carcinoma* OR neoplasia OR neoplasm*))

**PsychInfo 45 (18)**

TI ("quality of life" OR hrqol) OR (DE "Quality of Life" OR DE "Client Satisfaction" OR TI ("quality of life" OR hrqol OR "patient satisfaction" OR well-being) OR AB ("quality of life" OR hrqol OR "patient satisfaction" OR well-being)) AND (DE "Questionnaires" OR DE "Measurement" OR TI (assess* OR evaluat* OR measure* OR determin*) OR AB (assess* OR evaluat* OR measure* OR determin*)) OR TI ("Prostate Cancer Index" OR EORTC OR QLQ-C30 OR EORTC OR PR-25 OR EPIC OR SF-36) OR TI ("Prostate Cancer Index" OR EORTC OR QLQ-C30 OR EORTC OR PR-25 OR EPIC OR SF-36) AND (DE "Survivors" OR TI surviv* OR AB surviv*) AND (TI ('long term' OR longterm OR ((yr OR year*) N3 after) OR ((yr OR year*) N6 surviv*)) OR AB ('long term' OR longterm OR ((yr OR year*) N3 after) OR ((yr OR year*) N6 surviv*))) AND TI ((prostate OR prostatic) N3 (cancer OR adenocarcinoma* OR carcinoma* OR neoplasia OR neoplasm*)) OR AB ((prostate OR prostatic) N3 (cancer OR adenocarcinoma* OR carcinoma* OR neoplasia OR neoplasm*))

**Cochrane Library 141 (112)**

((prostate or prostatic) near/3 (cancer or adenocarcinoma* or carcinoma* or neoplasia or neoplasm*)):ti,ab,kw (Word variations have been searched) AND surviv*:ti,ab,kw and ('long term' or longterm or ((yr or year*) near/3 after) or ((yr or year*) near/6 surviv*)):ti,ab,kw (Word variations have been searched) AND ("quality of life" or hrqol):ti or ("Prostate Cancer Index" or EORTC or QLQ-C30 or EORTC or PR-25 or EPIC or SF-36):ti,ab,kw or ("quality of life" or hrqol or "patient satisfaction" or well-being) and (assess* or evaluat* or measure* or determin*):ti,ab,kw (Word variations have been searched)

**Pubmed 18 (10)**

Search (((((prostate[Title/Abstract] OR prostatic[Title/Abstract]) AND (cancer[Title/Abstract] OR adenocarcinoma[Title/Abstract] OR adenocarcinomas[Title/Abstract] OR carcinoma[Title/Abstract] OR carcinomas[Title/Abstract] OR neoplasia[Title/Abstract] OR neoplasm[Title/Abstract] OR neoplasms[Title/Abstract])) AND ((survivor[Title/Abstract] OR survivors[Title/Abstract] OR survived[Title/Abstract] OR surviving[Title/Abstract]) AND ("long term"[Title/Abstract] OR longterm[Title/Abstract]))) AND (("quality of life"[Title] OR hrqol[Title]) OR "Prostate Cancer Index"[Title/Abstract] OR EORTC[Title/Abstract] OR QLQ-C30[Title/Abstract] OR EORTC[Title/Abstract] OR PR-25[Title/Abstract] OR EPIC[Title/Abstract] OR SF-36[Title/Abstract] OR (("quality of life"[Title/Abstract] OR hrqol[Title/Abstract] OR "patient satisfaction"[Title/Abstract] OR well-being[Title/Abstract]) AND (assessment[Title/Abstract] OR assessed[Title/Abstract] OR measurement[Title/Abstract] OR measured[Title/Abstract] OR evaluation[Title/Abstract] OR evaluated[Title/Abstract] OR determination[Title/Abstract] OR determined [Title/Abstract]))))) AND (((inprocess[sb])) OR (publisher[sb] NOT pubstatusnihms NOT pubstatuspmcsd NOT pmcbook))

**Web of Science 704 (390)**

TS=((prostate OR prostatic) NEAR/3 (cancer OR adenocarcinoma* OR carcinoma* OR neoplasia OR neoplasm*)) AND TS=(surviv* AND ('long term' OR longterm OR ((yr OR year*) NEAR/3 after) OR ((yr OR year*) NEAR/6 surviv*))) AND TI=("quality of life" OR hrqol) OR TS=("Prostate Cancer Index" OR EORTC OR QLQ-C30 OR EORTC OR PR-25 OR EPIC OR SF-36) OR TS=(("quality of life" OR hrqol OR "patient satisfaction" OR well-being) AND (assess* OR evaluat* OR measure* OR determin*))

| B: Main findings of disease PC specific symptoms in RCTs | | | |
| --- | --- | --- | --- |
| Comp.: | **Study** | **Key Findings** | **Potential Limitation(s)** |
| S1^a^ | Donovan,  J L / 2016[37] | Comparison: AS vs. RP vs. EBRT, follow-up time^b^: 5-6 years, mean age^c^: 62 years  *Urinary Incontinence:*  - In general, RP had the greatest negative effect on incontinence and PC survivors treated with RP remained worse over time compared to PC survivors on AS or treated with EBRT  - Significant differences (p<0.001) in means over 6 years follow-up time between intervention groups for: ICIQ incontinence score, ICIQ incontinence problem scale, EPIC item: ≥1 pad per day, EPIC urinary score, ICSmaleSF urinary incontinence score, ICSmaleSF voiding score, ICSmale effect of urinary symptoms on QoL and ICSmaleSF nocturia item  - At 5 years scores across intervention groups were nearly similar  *Sexual Function:*  - RP had the greatest effect on erectile dysfunction and PC survivors treated with RP remained worse at all time points compared to PC survivors on AS or treated with EBRT  - Significant differences (p<0.001) in means over 6 years follow-up time between intervention groups for: Epic item erection firmness, EPIC problem with erectile dysfunction, EPIC sexual functions core, EPIC sexual bother score, EPIC sexual quality of life  *Bowel Function:*  - Bowel functions and bother scores as well as the effect of bowel habits were unchanged for PC survivors treated with RP or AS; Scores were worse for PC survivors treated with RT compared to the other intervention groups at all time points  - Significant differences (p<0.001) in means over 6 years follow-up time between intervention groups for: EPIC bowel function score, EPIC bowel bother score, EPIC item: loose stools, EPIC item: faecal incontinence, EPIC item: bloody stools, EPIC item: bowel habits |  |
| S1 | Giberti, C/ 2009[41] | Comparison: RP vs. BT, follow-up time^b^: 5 years, mean age^c^: 65.3 years  - No significant differences were observed among intervention groups in measures of PC specific symptoms | - Sample size <100 in both study arms  - No intention to treat analyses |
| S2 | Brundage, M/ 2015[36] | Comparison: ADT vs. ADT + EBRT, follow-up time^2^: 5-8 years, median age^3^: 69.7 years  - No signiﬁcant between-arm differences in any of the PC specific symptom scales at any time point 5+ years after diagnosis | - Sample size <100 in both study arms  - Only results on physical and role functioning were reported for this follow-up time |
| *Comp.* Comparison group  S1: HRQoL by primary intervention in long-term survivors with localized PC; S2: HRQoL by intervention in long-term survivors with locally advanced PC; S3: HRQoL  by intervention in long-term survivors with localized or locally advanced PC  Studies were ordered by stage information and within each group alphabetically  As potential limitation following criteria were considered: (1) sample size 100 per study arm for studies using EORTC-C30 and 70 for studies using SF-36 (2)  randomization (3) intention to-treat analyses (4) reporting of results appropriate  ^a^Inlcusion of PC survivors with disease progression  ^b^Time since randomization  ^c^Age at randomization | | | |

| C: Main findings of disease PC specific symptoms in observational studies | | | |
| --- | --- | --- | --- |
| Comp. | **Study** | **Key Findings** | **Potential Limitation(s)** |
| S1^a^ | Thong, M S/ 2010[47] | Comparison: AS vs. EBRT, follow-up time^b^: 7.8 years, mean age^d^: 75.8 years  - PC survivors treated with EBRT reported significantly (p<0.001) poorer bowel function and were more bothered by their bowel function  - No significant differences in urinary and bother function  - PC survivors treated with EBRT reported significantly more problems related to *“to getting and maintaining an erection in the last two weeks”*  Comparison: AS or EBRT vs. controls from the general population, follow-up time^b^: 7.8 years, mean age^d^: 75.8 years  - PC survivors of both intervention arms reported significantly poorer urinary function, and higher urinary and bowel bother  - PC survivors treated with EBRT reported significantly more problems related to *“to getting and maintaining an erection in the last two weeks”* and significantly lower scores for bowel function than controls from the general population | - No baseline data available |
| S2 | Namiki, S/ 2011[44] | Comparison: RP vs. EBRT, follow-up time^b^: 5 years, mean^e^: 69.5 years  - Patterns of alterations over time in intervention groups were different in urinary function (p<0.001), urinary bother (p=0.042), sexual function (p<0.001) and sexual bother (p<0.001) whereas survivors treated with RP had lower scores in all domains (=were worse) | - Sample size <70 in all study arms  - (Repeated ANOVA-tests: only changes over time are shown)  - No confounding control  - No adjustment for attrition error |
| S3^a^ | Berg, A/ 2007[35] | Comparison: EBRT + ADT/clinical progression vs. controls from the general population, follow-up time^b^: 10-16 years, median age^e^: 66 years  - PC survivors scored lower on sexual function domains and overall sexual satisfaction but similar on sexual problem assessment  Comparison: EBRT + ADT/clinical progression vs. EBRT, follow-up time^b^: 10-16 years, median age^e^: 66 years  - PC survivors treated additionally with ADT/clinical progressed reported poorer sexual function | - Sample size <100 in all study arms  - No confounding control  - No significance statistical test  -No adjustment for attrition error |
| S3^a^ | Fransson, P/ 2008[38] | Comparison: EBRT vs. controls from the general population, follow-up time^b^: 8 years, mean age^d^: 78.1 years  - Significant different (p<0.05) worse mean for PC survivor in: limitations in daily activities caused by urinary symptoms, starting problems urgency and pain while urinating  Comparison: EBRT vs. controls from the general population, follow-up time^b^: 15 years, mean age^e^: 78.1 years  - Significant different (p<0.05) worse mean for PC survivor in: limitations in daily activities caused by urinary symptoms, incontinence, stress incontinence and pain while urinating | - Sample size <100 in study arms  - No confounding control  - No adjustment for attrition error |
| S3 | Fransson, P/ 2009[39] | Comparison: EBRT vs. WW, follow-up time^c^: 10 years, median age^d^: 78 years  - PC survivors treated with EBRT had significantly (p=0.011) more problems with sexual bother and a weaker urinary stream  - No significant differences were reported for bowel problems | - Sample size <100 in both study arms |
| S3 | Johnstone, P A S/ 2000[42] | Comparison: EBRT (plus ADT) vs. controls from the general population, follow-up time^c^: 13.9 years, median age^d^: 80 years  - PC survivors reported more problems in: sexual function, sexual bother, urinary function, urinary bother, bowel function and bowel bother | - Sample size <70 in study arm  - No statistical significance test performed  - No confounding control  - No baseline data available |
| S3 | Namiki, S/ 2014[45] | For PC specific symptoms scales only longitudinal data reported for one intervention (RP) reported. | - Sample size <70 in study arms  - No adjustment for attrition error |
| S3^a^ | Shinohara, N/ 2013[46] | Comparison: EBRT vs. RP, localized and locally advanced PC, follow-up time: 5 years, mean/median age: 68 years  - PC survivors treated with RP reported significantly (p<0.05) worse urinary and sexual function | - Sample size <70 in all study arms  - No adjustment for attrition error  - No confounding control |

| Continuation Table C: Main findings of disease PC specific symptoms in observational studies | | | |
| --- | --- | --- | --- |
| Comp. | **Study** | **Key Findings** | **Potential Limitation(s)** |
| X | Galbraith, M E/ 2005[40] | Comparison: EBRT – LD^g^, EBRT – C^g^ vs. WW, follow-up time^c^: 5.5 years, age^d^: average 69.7 years  - PC survivors treated with EBRT-C reported significantly (p<0.01) less symptoms with urinary problems than PC survivors on WW  - PC survivors treated with RP reported significantly (p<0.05) less symptoms for gastrointestinal problem than PC survivors treated with EBRT-C^4^  - PC survivors treated with EBRT-MB^4^ reported significantly (p<0.05) less symptoms for sexual problems than PC survivors on WW | - Sample size <70 in all study arms  - No confounding control  - For growth curve analyses plots are printed badly, so it cannot be distinguished between intervention arms  - For comparisons at specific time points it is not explained which statistical tests was used  - P-values are not shown for all comparisons, not explained for which reasons some results are not shown  - No adjustment for attrition error |
| *Comp.* Comparison group  S1: HRQoL by primary intervention in long-term survivors with localized PC; S2: HRQoL by intervention in long-term survivors with locally advanced PC; S3: HRQoL by intervention in long-term survivors with localized  or locally advanced PC; X: No assignment possible as study revealed no information about cancer stage  Studies were ordered by stage information and within each group alphabetically  As potential limitations, the following criteria were considered: (1) sample size 100 per study arm for studies using EORTC-C30 and 70 for studies using SF-36 70 (2) adjustment for attrition error (3) statistical significance tests performed (4) adjustment for attrition error (only prospective cohort studies) (5) baseline data available (6) reporting of results appropriate  Definition of clinically meaningful difference: EORTC QLQ-C30: min. 10 points difference; SF-36: min. 5 points difference in general health dimension, min 6.5 points in physical dimension, 7.9 points in mental  health dimension  ^a^Inlcusion of PC survivors with disease progression  ^b^Time since diagnosis  ^c^Time since enrolment in study  ^d^Age at survey  ^e^Age at enrollment in study  ^f^Not reported, but clinically meaningful difference  ^g^EBRT-LD — Low-dose mixed-beam radiation, EBRT-C — Conventional radiation | | | |
